# Predictive biomarkers of anti-PD-1/PD-L1 therapy in NSCLC

**DOI:** 10.1186/s40164-021-00211-8

**Published:** 2021-03-02

**Authors:** Mengke Niu, Ming Yi, Ning Li, Suxia Luo, Kongming Wu

**Affiliations:** 1 Department of Medical Oncology, The Affiliated Cancer Hospital of Zhengzhou University & Henan Cancer Hospital, Zhengzhou, 450008 China; 2 Department of Oncology, Tongji Hospital of Tongji Medical College, Huazhong University of Science and Technology, Wuhan, 430030 China

**Keywords:** NSCLC, Immunotherapy, Biomarkers, Anti-PD-1/PD-L1 therapy, Efficacy prediction

## Abstract

Immunotherapy, especially anti-programmed cell death protein 1/programmed cell death ligand 1 (PD-1/PD-L1) treatment has significantly improved the survival of non-small cell lung cancer (NSCLC) patients. However, the overall response rate remains unsatisfactory. Many factors affect the outcome of anti-PD-1/PD-L1 treatment, such as PD-L1 expression level, tumor-infiltrating lymphocytes (TILs), tumor mutation burden (TMB), neoantigens, and driver gene mutations. Further exploration of biomarkers would be favorable for the best selection of patients and precisely predict the efficacy of anti-PD-1/PD-L1 treatment. In this review, we summarized the latest advances in this field, and discussed the potential applications of these laboratory findings in the clinic.

## Background

Lung cancer has a high incidence rate worldwide and is the main cause of cancer deaths [1]. The 5-year survival rate varies in different regions [2]. Non-small cell lung cancer (NSCLC) accounts for approximately 80–85% of all lung cancers [3, 4]. Recently, the anti-programmed cell death protein 1/programmed cell death ligand 1 (PD-1/PD-L1) treatment has substantially changed the treatment patterns of NSCLC. The anti-PD-1/PD-L1 treatment with or without platinum-based chemotherapy has become the first-line strategy for NSCLC without driver gene mutations [5].

The immune system can specifically recognize the expression of tumor-specific antigens and eliminate tumor cells [6]. Alterations in effector cell signal transduction molecule (T cell receptor/CD3), the levels of tumor antigens, the maturation of antigen-presenting cells (APC), tumor-derived soluble factors such as vascular endothelial growth factor (VEGF), transforming growth factor-β (TGF-β), and IL-10 propel tumor immune escape [7–11]. PD-1 and PD-L1 are type I transmembrane proteins[12]. The interaction of PD-1 and PD-L1 leads to the phosphorylation of the cytoplasmic immunoreceptor tyrosine-based inhibitory motif (ITIM) and the immunoreceptor tyrosine-based switch motif (ITSM) and recruits Src homology 2 domain containing phosphatases 1/2 (SHP1/2) [13]. The recruitment of SHP1/2 inhibits the activation of T cells [14]. SHP1/2 and their downstream inhibitory signaling pathways suppress the activation of phosphatidylinositol 3-kinase (PI3K)/protein kinase B (AKT) and mitogen-activated protein kinase (MAPK) [15, 16].

The anti-PD-1/PD-L1 treatment blocks the interaction of PD-1 and its ligands, interferes with inhibitory signal transduction, restores the vitality of T cells, and thereby restarts the anti-tumor immune effect [17, 18]. NSCLC has high level of heterogeneity. The heterogeneity of molecular immune subtypes and immune microenvironment results in the differences in the efficacy of PD-1/PD-L1 inhibitors [19]. The low response rate to PD-1/PD-L1 inhibitors hinders the clinical application [20]. Therefore, it is urgent to find reliable biomarkers to effectively predict the efficacy of PD-1/PD-L1 inhibitors. In this review, we summarized the latest advances in the predictive biomarkers of anti-PD-1/PD-L1 therapy in NSCLC.

## Tumor feature related biomarkers

### PD-L1 expression level

**Fig. 1:**
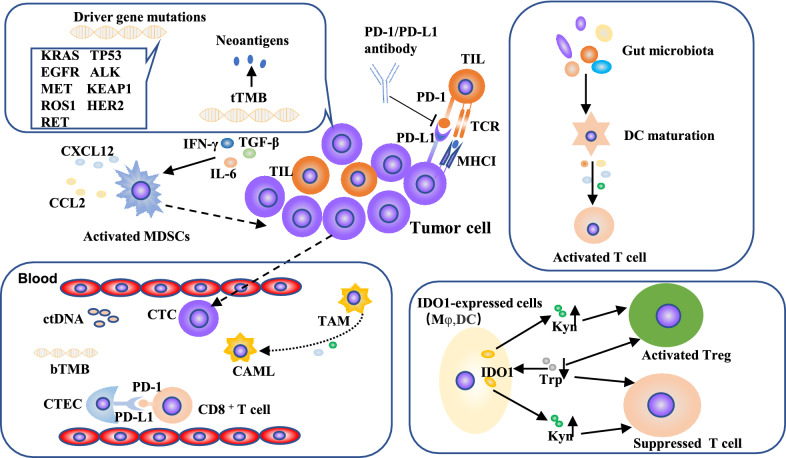
Predictive biomarkers of anti-PD-1/PD-L1 therapy in NSCLC. First, increased PD-L1 level is an indicator of the pre-existed anti-tumor immune response which is positively correlated to response rate to anti-PD-1/PD-L1 treatment. Second, TIL is the effectors of anti-tumor immune response, which could be boosted by PD-1/PD-L1 inhibitors. Besides, TMB and neoantigens determine cancer immunogenicity which is the basis of anti-tumor response. In addition, multiple other factors such as suppressive immune cells, driver gene mutations, gut microbiota, tumor metabolites such as IDO1 also participate in anti-tumor immunity and affect the efficacy of anti-PD-1/PD-L1 therapies

A known mechanism for PD-1/PD-L1 to promote tumor immune escape is adaptive immune resistance [21]. Multiple clinical trials have been performed to evaluate the relationship between the expression of PD-L1 on tumor cells and the response rate to PD-1/PD-L1 inhibitors (Fig. 1). The high level of PD-L1 expression heralds the potential benefit of anti-PD-1/PD-L1 treatment [22, 23]. In the phase I KEYNOTE-001 study, among patients who had previously treated with anti-PD-1 therapy, patients with PD-L1 tumor proportion score (TPS) ≥ 50% had a median overall survival (OS) of 15.4 months (95% CI: 10.6–18.8 months) (Table 1) and the 5-year OS rate was 25.0%; while in the PD-L1 TPS 1%-49% group and PD-L1 TPS ≤ 1% group, the median OS were 8.5 months (95% CI: 6.0–12.6 months) and 8.6 months (95% CI: 5.5–10.6 months), and the 5-year OS rates were 12.6% and 3.5%, respectively [22]. In the multicenter, single-arm, open-label phase II clinical trial (PePS2), the incidence of durable clinical benefit (DCB) in the PD-L1 TPS ≥ 50% group was 53% (95% CI: 30–75%) (Table 1), while the PD-L1 TPS 1–49% group and PD-L1 TPS ≤ 1% group were 47% (95% CI: 25–70%) and 22% (95% CI: 11–41%) [23]. In KEYNOTE-024 study, pembrolizumab treatment lengthened the survival time of NSCLC patients with PD-L1 TPS ≥ 50%, relative to platinum-based chemotherapy (HR = 0.63, 95% CI: 0.47–0.86, *p* = 0.002) [24] (Table 1). However, only evaluating PD-L1 level can’t accurately select patients. Other studies showed that regardless of the level of PD-L1 expression, renal cell cancer (RCC) or NSCLC patients with anti-PD-1/PD-L1 treatment had survival benefits [25, 26]. The outcome of PD-1/PD-L1 blockade therapy was also determined by other characteristics including the immune status, the activity of the tumor-infiltrating T cells and the sensitivity of cancer cells to T cells [27]. Therefore, clinical decisions should be made carefully based on the results of PD-L1 expression.

**Table 1:** Predictive biomarkers of anti-PD-1/PD-L1 therapy in NSCLC

	Biomarkers		The predictive effect of biomarkers		Reference
Tumor feature		PD-L1 expression level	15.4 months, 95%CI: 10.6–18.8 months (median OS) 53%, 95%CI: 30–75% (DCB) HR: 0.63, 95%CI: 0.47–0.86 (OS)		[22–24]
		TMB	tTMB 29 vs. 6% (ORR) HR: 0.62, 95%CI: 0.38–1.00 (PFS) HR: 0.58, 97.5%CI: 0.41–0.81 (PFS) bTMB HR: 0.39, 95%CI: 0.18–0.84 (PFS)		[28, 30–32]
		Neoantigens	HR: 0.23, 95%CI: 0.09–0.58 (median PFS) 92 vs. 11% (DCB)		[34, 42]
		Driver gene mutations	*KRAS* OR: 1.51; 95%CI: 1.17–1.96 (ORR) *TP53* HR: 0.32, 95%CI: 0.16–0.63 (PFS) *EGFR* 5.3 months, 95%CI: 1.3–12.4 months (median PFS) *ALK* 0.6 months, 95% CI: 0.2–2.1 months (PFS) *MET* 17%, 95%CI: 6%-36% (ORR) *NFE2L2/KEAP1* 22.52 months vs. 12.89 months (median OS)		[46, 47, 51, 53, 55, 61]
		miRNA	HR: 0.45, 95%CI: 0.25–0.76 (median PFS) HR: 0.39, 95%CI: 0.15–0.68 (median OS)		[71]
Tumor microenvironment		TIL	HR: 0.954, 95%CI: 0.965–0.983 (DFS) HR: 0.965, 95%CI: 0.931–1.001 (OS)		[78]
Biomarkers in peripheral blood		CAMLs	HR: 2.5, 95%CI: 1.1–5.8 (PFS) HR: 3.5, 95%CI: 1.3–9.6 (OS)		[109]
		CTECs	5 months, 95%CI: 3.9–6.1 months (median PFS)		[116]
		Other peripheral blood cells	NLR HR: 1.44, 95%CI: 1.26–1.65 (median PFS) HR: 2.86, 95%CI: 2.11–3.87 (median OS)		[119]
Other		Gut microbiota	HR: 4.2, 95%CI: 1.42–12.3 (PFS)		[130]
		Patients clinical characteristics	Gender male: HR: 0.76, 95%CI: 0.64–0.91 female: HR: 0.44, 95%CI: 0.25–0.76 (OS) Smoking 36 vs. 26 vs. 14% (current smokers vs. former smokers vs. non-smokers) PIOS HR: 0.469, 95%CI: 0.295–0.747 (median PFS) HR: 0.539, 95%CI: 0.317–0.918 (median OS)		[141, 142, 144]

*PD-L1* programmed cell death ligand 1, *CI* confidence interval, *OS* overall survival, *DCB* durable clinical benefit, *TMB* tumor mutational burden, *ORR* objective response rate, *HR* hazard ratio, *PFS* progression-free survival, *KRAS* kirsten rat sarcoma 2 viral oncogene homolog, *OR* odds ratio, *TP53* tumor protein p53, *EGFR* epidermal growth factor receptor, *ALK* anaplastic lymphoma kinase, *MET* mesenchymal epithelial transition, *KEAP1* kelch-like ECH-associated protein 1, *NFE2L2* nuclear factor erythroid-2-related factor-2, *TIL* tumor-infiltrating lymphocyte, *DFS* disease-free survival, *CAMLs* circulating cancer-associated macrophage-like cells, *CTECs* circulating tumor endothelial cells, *NLR* neutrophil to lymphocyte ratio, *PIOS* patras immunotherapy score

### Tumor mutation burden (TMB)

Whole-exome sequencing (WES) and sequencing of cancer gene panels (CGPs) are used to measure deoxyribonucleic acid (DNA) mutations in tumor tissue [28]. The tumor tissue TMB (tTMB) is positively correlated with tumor neoantigen load (Fig. 1) [29]. Multiple retrospective studies showed that tTMB was closely associated with the efficacy of PD-1/PD-L1 inhibitors and patient’s prognosis. In KEYNOTE-158, for patients treated with pembrolizumab, the tTMB-High group had a higher objective response rate than the non-tTMB-High group (29 vs. 6%) (Table 1) [30]. In CHECKMATE-026, patients with high tTMB receiving nivolumab treatment had a longer progression-free survival (PFS) (9.7 vs. 5.8 months; HR = 0.62, 95% CI: 0.38–1.00) (Table 1) and higher response rate (47 vs. 28%) than patients receiving chemotherapy [31]. Similarly, the results of CHECKMATE-227 showed that in patients with high tTMB, nivolumab plus ipilimumab group had a longer PFS than chemotherapy group (7.2 vs. 5.5 months; HR = 0.58, 97.5% CI: 0.41–0.81, *p* < 0.001) [32] (Table 1).

Blood TMB (bTMB) is discovered as a new and less invasive alternative, which is measured by detecting plasma cell-free DNA (Fig. 1) [28]. bTMB is positively correlated to tTMB [28]. Compared with bTMB < 6 subgroup, the bTMB ≥ 6 subgroup had higher objective response rate (39.3 vs. 9.1%) and longer PFS (HR = 0.39, 95% CI: 0.18–0.84, *p* = 0.01) (Table 1) [28]. However, the relationship between bTMB and patient’s survival showed a non-linear correlation [33]. For patients treated with PD-L1 inhibitors, the bTMB-High (≥ 14 mutations/Mb) and bTMB-Low (≤ 7 mutations/Mb) subgroups had longer PFS and OS than bTMB-Medium (8–13 mutations/Mb) subgroup [33]. The positive correlation between baseline circulating tumor DNA (ctDNA) and bTMB score explained the better prognosis of the bTMB-low patients [33]. In addition, compared with patients of bTMB-Medium, bTMB-low patients had longer response duration and higher stable disease rate [33]. In general, hypermutation promoted the production of tumor neoantigens, enhanced tumor immunogenicity and improved the response rate to PD-L1 inhibitors [34].

### Neoantigens

Neoantigens are derived from somatic mutation [35], which bind to major histocompatibility class I (MHCI) and are expressed on the surface of cancer cells. Neoantigens endow the tumor with high immunogenicity and induce anti-tumor immune response (Fig. 1) [36]. Neoantigens are released by tumor cells and captured by professional APC, and then the effector T cells targeting cancer specific antigens are activated [37]. Activated T cells migrate and infiltrate into tumor bed, specifically recognize the antigens on tumor cells and kill cancer cells [37]. The tumor clones with potent immunogenicity are eliminated, and the cancer cells with weak immunogenicity escape immune surveillance [38]. Many studies proved that anti-PD-1/PD-L1 therapy combined with radiotherapy or oncolytic virus increased the release of neoantigens and amplified the specific immune response [39–41]. Compared with no durable clinical benefit (NDB) patients, DCB patients had higher burden of candidate neoantigens. High candidate neoantigen burden was associated with improvement in PFS (HR = 0.23, 95%CI: 0.09–0.58, *p* = 0.002) [42] (Table 1). The efficacy of immunotherapy was not only related to the quantity of neoantigens, but also related to the quality of neoantigens [43]. High-quality neoantigens especially clonal neoantigens, could bind to multiple HLA alleles [43]. The clonal neoantigens promoted the activation and infiltration of neoantigen reactive T cells expressing high level of PD-1, and tumors enriched clonal neoantigens were more sensitive to PD-1 blockers [34]. The incidence rate of DCB in patients with high mutation burden and low neoantigen subclonal fraction was higher than patients with high subclonal neoantigen fraction or low clonal neoantigen burden (92 vs. 11%) [34] (Table 1). Immune elimination of neoantigen-containing tumor cell subpopulations and genetic events such as chromosomal deletions or loss of heterozygosity in tumor cells lead to the loss of neoantigens, which contribute to the emergence of acquired resistance to anti-PD-1/PD-L1 treatment [44].

### Driver gene mutations

Next-generation sequencing (NGS) is widely used for tumor genome analysis [45]. The gene alterations detected by targeting NGS may herald the response rate to PD-1/PD-L1 inhibitors (Fig. 1) [45]. Kirsten rat sarcoma 2 viral oncogene homolog (*KRAS*) mutation status was positively correlated with PD-L1 expression [46]. In addition, *KRAS* mutant-type tumors had more TILs and higher TMB, which presented the inflammatory phenotype of adaptive immune resistance and increased immunogenicity [46]. Compared with *KRAS* wild subgroup, *KRAS* mutated subgroup had a higher objective response rate (odds ratio = 1.51, 95% CI: 1.17–1.96, *p* = 0.002) (Table 1) [46]. *TP53*-mutated tumors had high PD-L1 expression and CD8^+^ T cell density [47]. Patients with *TP53* mutations and no serine/threonine kinase 11 (*STK11*) or epidermal growth factor receptor (*EGFR*) co-mutations had higher response rate and longer PFS to anti-PD-1 therapy (HR = 0.32, 95%CI: 0.16–0.63, *p* < 0.001). Pathways related to immune cell cytotoxicity, T cell chemotaxis, antigen processing were upregulated in this tumor subtype [47]. *EGFR* with exon 19 deletion, L858R mutation and T790M mutation upregulated the expression of PD-L1, which attenuated cytotoxicity of lymphocytes and induced T-cell exhaustion through PD-1/PD-L1 axis [48–50]. Among patients who treated with anti-PD-1 therapy, patients with *EGFR* mutations had worse prognosis (median PFS: 5.3 months, 95% CI: 1.3–12.4 months) [51]. The anaplastic lymphoma kinase (*ALK*)-rearranged upregulated PD-L1 expression and promoted tumor immune escape [52]. However, *ALK*-mutated patients who treated with anti-PD-1 therapy presented worse PFS than patients with *EGFR* mutations (*ALK:* 0.6 (95% CI: 0.2–2.1) months, *EGFR:* 1.8 (95% CI: 1.2–2.1) months), suggesting that PD-L1 expression was not a reliable biomarker for immunotherapy for patients with *ALK* rearrangement [53]. The mesenchymal epithelial transition (*MET*) exon 14 skipping alterations occur in 3%-4% of lung cancers [54]. A large proportion of lung cancer cells with *MET* exon 14 alterations expressed PD-L1 [55]. Lung cancer patients with *MET* exon 14 mutations responded modestly to single-agent or combination immune checkpoint inhibitors (objective response rate: 17%, 95% CI: 6%-36%) [55], and didn’t seem to benefit from immunotherapy [56]. Kelch-like ECH-associated protein 1 (*KEAP1*) somatic mutations promoted tumorigenesis and reduced therapeutic sensitivity by activating the *KEAP1*/nuclear factor erythroid-2-related factor-2 (*NFE2L2*) stress response pathway [57–60]. *NFE2L2*/*KEAP1* mutations were associated with high TMB and PD-L1 expression, and the efficacy of immunotherapy was better in patients with *NFE2L2*/*KEAP1* mutations than other treatments (median OS: 22.52 months vs. 12.89 months, *p* = 0.0034) [61]. The mutation status of other rare driver genes such as *ROS1*, *HER2*, *RET* may also affect the response to PD-1/PD-L1 inhibitors [62, 63].

### Inflammation related genes

Some expression signatures reflect the inflammatory state of tumors, such as genes related to T cell activation, chemokine expression, and adaptive immune resistance (Fig. 1) [64, 65]. Patients with significantly elevated inflammatory profile scores tended to be sensitive to PD-1/PD-L1 inhibitors. Compared with non-responders, responders had significantly higher inflammation signature scores [65]. In addition, inflammation scores was correlated with epithelial-mesenchymal transition (EMT) scores. Thompson’s study showed that the combination of EMT phenotypic feature scores and inflammation gene scores increased the accuracy of prediction [65]. Therefore, it is predicted that reversal of EMT may improve the resistance to anti-PD-1/PD-L1 therapy [65]. Further study found that in the same NSCLC cohort, the eight genes associated with antigen processing machinery (APM) scores could more effectively predict the efficacy than inflammation scores [66]. Also, our previous study indicated that some immune response-related signatures related to the efficacy of immune checkpoint inhibitor in lung adenocarcinoma [4].

### microRNA(miRNA)

MiRNA modifies the expression of target genes by regulating protein translation [67]. miRNA dysregulation is closely associated with carcinogenesis and can promote or suppress cancer by targeting a group of genes (Fig. 1) [68]. In addition, miRNA regulates anti-tumor immunity. Some miRNAs interfere with antigen processing and presentation, upregulate human leukocyte antigen (HLA)-G expression and downregulate natural killer group 2, member D (NKG2D) ligand to form immune escape [69]. Circular RNA circ-CPA4 upregulated PD-L1 expression in NSCLC cells by downregulating let-7 miRNA [70]. 10-high expressed miRNAs (miR-93, miR-138-5p, miR-200, miR-27a, miR-424, miR-34a, miR-28, miR-106b, miR-193a-3p, miR-181a) were found in responders treated with anti-PD-1 treatment, and associated with significantly improved PFS and OS (median PFS: 6.25 months vs. 3.21 months, HR = 0.45, 95% CI: 0.25–0.76; median OS: 7.65 months vs. 3.2 months, HR = 0.39, 95% CI: 0.15–0.68) (Table 1) [71].

## Tumor microenvironment related biomarkers

### Tumor-infiltrating lymphocyte (TIL)

Previous reports shown that PD-L1 expression was significantly associated with intratumoral T cells infiltration in NSCLC [72]. The transcription factor thymocyte selection-associated high mobility group box gene (*TOX*) in tumor-infiltrating CD8^+^ T cells promotes T cell exhaustion by upregulating the expression of immune checkpoint proteins PD-1, T cell immunoglobulin and mucin-domain containing-3 (TIM-3) [73], T cell immunoglobulin and ITIM domain (TIGIT) [74], and cytotoxic T lymphocyte antigen 4 (CTLA-4), thereby attenuates the outcome of anti-PD-1 therapy (Fig. 1) [75]. Based on PD-L1/TIL status, NSCLC tumor immune microenvironments were divided into type I (PD-L1^+^, TIL^+^), type II (PD-L1^−^, TIL^−^), type III (PD-L1^+^, TIL^−^) and type IV (PD-L1^−^, TIL^+^) [76]. The difference in clinical factors related to different tumor immune microenvironment types determines the patient selection for combination immunotherapies [76]. Type I tumors benefit greatly from anti-PD-1/PD-L1 therapy. However, Type III tumors are resistant to anti-PD-1/PD-L1 monotherapy, which could be reversed by the combining adjuvant therapy to recruit T cells into tumor bed [77]. The proportion of CD8^+^ cells among the overall population of CD3^+^ TILs has a close relationship with anti-PD-1/PD-L1 treatment outcomes. It has been shown that High CD8-to-CD3 ratio was positively correlated with disease-free survival (DFS) and OS (DFS: HR = 0.954, 95%CI: 0.965–0.983, *p* = 0.002; OS: HR = 0.965, 95%CI: 0.931–1.001, *p* = 0.057) (Table 1) [78]. The early proliferation of CD8^+^ T cells after anti-PD-1 therapy heralded a good clinical response to anti-PD-1 therapy [79]. T cell receptor (TCR) is expressed on the surface of T cells and composed of α chains and β chains, which form diversity and specificity through somatic DNA rearrangement [80]. TCR binds to MHC/antigen short peptide complex and triggers immune response (Fig. 1) [81]. The TCR β chain complementarity determining region 3 of PD-1^+^ CD8^+^ T cells was sequenced by multiplex PCR. The diversity of TCR before anti-PD-1/PD-L1 treatment heralded a better survival outcome (6.4 vs. 2.5 months, HR = 0.39, 95% CI: 0.17–0.94, *p* = 0.021), and the clonality of TCR after treatment also heralded clinical benefit (7.3 vs. 2.6 months, HR = 0.26, 95% CI: 0.08–0.86, *p* = 0.002) [82].

Consolidation therapy with durvalumab after concurrent chemo-radiotherapy (cCRT) could significantly improve the overall survival and median progression-free survival of patients as compared with placebo group [83]. Radiotherapy stimulated anti-tumor immunity by promoting the release of tumor neoantigens and driving the immune attack of CD8 ^+^ TILs [84]. Post-cCRT PD-L1 upregulation might be in response to radiotherapy-related immune attack, which provided theoretical basis for the application of PD-L1 blockers following cCRT [85]. In addition, increased CD8 ^+^ TIL density after cCRT was associated with favorable survival [85].

### Suppressive immune cell

Tumor-infiltrating regulatory T lymphocytes (Tregs) express PD-L1, PD-L2 on the surface, which highly inhibit the activity of tumor-specific effector T cells [86]. Indoleamine 2,3-dioxygenase 1 (IDO1) induces T cells immune suppression and Treg hyperactivation by l-tryptophan (Trp) depletion and kynurenine (Kyn) accumulation in the tumor microenvironment (Fig. 1) [87]. Serum kyn/trp ratio may reflect the anti-PD-1 immune resistance mechanism [88]. Myeloid-derived suppressor cells (MDSCs) mainly play an immunosuppressive role in the tumor microenvironment [89]. Some inflammatory factors such as TGF-β, IFN-γ, and IL-6 drive the activation of MDSCs [90]. Chemokines such as C–C motif chemokine ligand 2 (CCL2) [91] and C-X-C motif chemokine ligand 12 (CXCL12) [92] recruit MDSCs to tumor sites. MDSCs inhibit the immune response of tumor-specific T cells by upregulated PD-L1 expression (Fig. 1) [93].

### Extracellular vesicles (EVs)

EVs are a collection of membrane-bound carriers, which carry lipids, proteins, and nucleic acids [94]. Budding inward through endosomal pathways to form exosomes and sprouting out of the plasma membrane to form microvesicles [95]. EVs bind to target cells and initiate signal transduction through receptor-ligand interactions or internalize through endocytosis [96]. EVs mediate cancer cell sensitivity to chemotherapy and radiotherapy, and are promising strategy in liquid biopsy for cancer diagnosis and predictive markers [97, 98]. The exchange of EVs between immune cells affects innate immunity and adaptive immunity [99]. Local dendritic cells (DCs) secreted-EVs could induce T cell activation [95]. EVs are key components in the microenvironment that bridge the communication between tumor cells and stromal cells [100]. By extracting EVs miRNAs from advanced NSCLC patients receiving anti-PD-1/PD-L1 therapy for sequencing analysis, a remarkable difference in the concentration of specific miRNAs between responders and non-responders was found [101]. As a non-invasive liquid biopsy, early detection of tumor-derived EVs may help to predict the efficacy of anti-PD-1/PD-L1 therapy [102–104].

## Biomarkers in peripheral blood

### Circulating cancer-associated macrophage-like cells (CAMLs)

Tumor associated macrophage (TAM) promotes the invasion characteristics of malignant cells by secreting growth factors and cytokines such as VEGF, MMP, TNF-α [105]. TAM and circulating tumor cells (CTC) migrate to the blood circulation through lymphatic or capillary barrier, which enhance tumor invasion and distant metastasis [106]. As a diffuse TAM (Fig. 1), the isolation of CAMLs from peripheral blood of various cancer patients may be evidence of tumor metastasis and neovascularization [107]. CAMLs were quantified by the CellSieve system using multiplex immunostaining [108]. CAMLs ≥ 50 μm was defined as giant CAMLs. The size of CAMLs after completion of CRT was related to disease progression and patient’s survival [109]. The presence of giant CAMLs before anti-PD-L1 maintenance therapy indicated a poor prognosis (median PFS: 8 months, HR = 2.5, 95% CI: 1.1–5.8, *p* = 0.025; median OS: 25 months, HR = 3.5, 95% CI: 1.3–9.6, *p* = 0.034) (Table 1). The tumor-stimulating effect of CAMLs may limit the efficacy of anti- PD-L1 therapy [109].

### PD-L1^+^ aneuploid circulating tumor endothelial cells (CTECs)

The aneuploidy of chromosome influences gene expression and determines tumor heterogeneity, which is closely related to the evolution of tumor [110–112]. CTECs, aneuploid CD31^+^ circulating tumor endothelial cells [113], are derived from aneuploid CD31^+^ tumor endothelial cells in tumor tissue and promote tumor angiogenesis [114, 115]. The PD-L1^+^ CTECs had morphological and karyotype changes after immunotherapy [116]. Anti-PD-1 could effectively eliminate haploid small CTECs, while relatively increase polyploid large PD-L1^+^ CTECs [116]. Patients with PD-L1^+^ CTECs subtype were resistant to anti-PD-1 treatment. The median PFS of patients with PD-L1^+^ CTECs was 5 months (95% CI: 3.9–6.1 months) (Table 1), which was shorter than that of patients without PD-L1^+^ CTECs (8 months, 95% CI: 4.9–11 months). It was speculated that the interaction of PD-L1 on CTECs with PD-1 on T cells inhibited the tumor-specific immune attack of CD8^+^ T cells and affected the efficacy of immunotherapy (Fig. 1) [116].

### Other peripheral blood cells

Among many indicators that reflect inflammation, the high neutrophil to lymphocyte ratio (NLR) heralded a poor prognosis in many malignant tumors [117, 118]. Multiple studies found that NSCLC patients with high NLR had low response rate to immune checkpoint inhibitors (ICIs) [119, 120]. A meta-analysis showed that patients with high NLR before ICIs therapy had poor prognosis (PFS: HR = 1.44, 95%CI: 1.26–1.65, *p* < 0.001; OS: HR = 2.86, 95%CI: 2.11–3.87, *p* < 0.001) (Table 1) [119]. Similarly, another retrospective study also verified the predictive value of NLR for anti-PD-1 treatment [120]. Lactate dehydrogenase (LDH) is an indicator of cancer-related inflammation [121]. According to the values of LDH and NLR, lung cancer patients were divided into 3 groups (good, 0 factors; intermediate, 1 factor; poor, 2 factors). Compared with the good group, the intermediate group and poor group were more easily resist to anti-PD-1/PD-L1 treatment [121]. In addition, NLR and LDH might be useful indicators for predicting irAEs [122]. Neutrophils were highly correlated with myeloid phenotype, which promoted lymphocyte depletion [123]. Tumor-infiltrating CD8^+^ T cells to neutrophils (CD8/PMN) ratio could distinguish responders treated with anti-PD-1 therapy [123]. Combining neutrophil antagonists improved immunotherapy outcomes [123]. Besides, the amount and activity of NK cells in responders were highly elevated [124].

## Gut microbiota

Gut microbiota has a symbiotic relationship with the host [125]. In addition to playing a barrier role in the gastrointestinal tract, microorganisms are related to the immune function of the plora [126]. Immune cells are activated through cross-reactivity between microbial proteins and tumor antigens [127]. DCs induce activated T cells outside the intestine, recognize tumor antigens and exert anti-tumor effect [127]. In addition, the microbial proteins translocate from the intestine to the blood circulation, trigger initial immunity in secondary lymphoid organs and induce the activation of T cells. T cells migrate to the tumor site and participate in immune surveillance (Fig. 1) [127]. The composition of microorganisms may affect the efficacy of PD-1 inhibitors [128]. A study showed that the fecal *Akkermansia muciniphila* could be detected in 69% (11/16) and 58% (23/40) of patients exhibiting partial response or stable disease, whereas it was detectable in 34% (15/44) of patients who progressed or died [129]. Gut microbiota profiles of fecal specimens could be assessed by 16S ribosome RNA gene sequencing. *Alipis putredinis*, *Prevotella copri* and *Bifidobacterium longum* were enriched in the responders, and *Ruminococcus_unclassified* was enriched in non-responders. Patients with higher microbiota diversity had significantly longer PFS (HR = 4.2, 95%CI: 1.42–12.3, *p* = 0.009) (Table 1) [130]. The microbiota associated with clinical benefit varies in different studies, which implied that the difference between diet, host genetics, lifestyle factors, and human species may contribute to the diversity of gut microbiome and further affect the efficacy of ICIs [131, 132].

The application of cumulative antibiotics (ATB) could reduce the diversity of gut microbiota and disrupt the microbial balance [133, 134], which significantly weakened the efficacy of PD-L1 inhibitors and affected survival outcomes (median PFS: 1.9 months, HR = 1.5, 95%CI: 1.0–2.2, *p* = 0.03; median OS: 7.9 months, HR = 4.4, 95%CI: 2.6–7.7, *p* < 0.01) [135]. A study indicated that proton pump inhibitor (PPI) affected the diversity of gut microbiota through gastric acid [136]. The data of the phase II POPLAR and phase III OAK trial showed that in the population of anti-PD-L1 therapy, patients treated with ATB or PPI had shorter OS (HR = 1.20, 95%CI: 1.04–1.39) (Table 1), and the application of PPI was significantly related to shorter PFS (HR = 1.26, 95%CI: 1.10–1.44) [137]. As a promising treatment method, fecal microbiome transplantation (FMT) could improve the diversity of gut microbiota and the efficacy of immunotherapy [138, 139].

## Patient's clinical characteristics

Factors such as genes, hormones contribute to the differences in immune response between males and females [140]. The differences may affect the efficacy of immunotherapy for male and female malignant tumors [140]. In a meta-analysis, by comparing the effects of anti-PD-1/PD-L1 plus chemotherapy and chemotherapy alone in men and women, it was found that the pooled OS-HRs were 0.76 (95% CI: 0.66–0.87) for men and 0.48 (95% CI: 0.35–0.67) for women [141]. Another meta-analysis showed that the pooled OS-HRs were 0.78 (95% CI: 0.60–1.00) in men and 0.97 (95% CI: 0.79–1.19) in women for anti-PD-1 alone, compared with 0.76 (95% CI: 0.64–0.91) in men and 0.44 (95% CI: 0.25–0.76) in women for anti-PD-1/PD-L1 plus chemotherapy [141] (Table 1). This implied that anti-PD-1 monotherapy may have a greater impact on men, and women may obtain greater survival benefits from the combination of anti-PD-1/PD-L1 and chemotherapy [141]. Nearly 80% of lung cancers are related to smoking. Exploratory analysis showed that among patients treated with anti-PD-1 treatment, current and former smokers had significantly higher overall response rate than non-smokers (36 vs. 26 vs. 14%) (Table 1) [142]. In addition, the increase of smoking years was associated with positive anti-PD-1 therapy response [143]. The patras immunotherapy score (PIOS) including the patient’s performance status (PS), body mass index (BMI), lines of treatment (LOT) and age was calculated through the formula (PS × BMI/LOT × age). Patients with high PIOS score had the best response to anti-PD-1 treatment (median PFS: 15 months vs. 5 months, HR = 0.469, 95% CI: 0.295–0.747; median OS: 32 months vs. 14 months, HR = 0.539, 95% CI: 0.317–0.918) (Table 1) [144].

## Conclusion

Anti-PD-1/PD-L1 treatment is a promising treatment strategy for NSCLC. However, there are still numerous patients who are difficult to benefit from anti-PD-1/PD-L1 treatment. Various biomarkers for predicting efficacy are being explored. In the present stage, PD-L1 expression is the most widely adopted biomarker in clinical practice. TMB, TIL and neoantigen are significantly correlated with the efficacy of anti-PD-1/PD-L1 therapy. Gut microbiota, inflammatory genes, and dysregulated miRNA play an important role in anti-tumor immune regulation. Combining of multiple biomarkers may increase the predictive robustness and guide the implementation of cancer precision medicine.

## Data Availability

Not applicable.

## Abbreviations

PD-1: Programmed cell death protein 1
PD-L1: Programmed cell death ligand 1
NSCLC: Non-small cell lung cancer
TILs: Tumor-infiltrating lymphocytes
TMB: Tumor mutational burden
TPS: Tumor proportion score
OS: Overall survival
PFS: Progression-free survival
KRAS: Kirsten rat sarcoma 2 viral oncogene homolog
EGFR: Epidermal growth factor receptor
ALK: Anaplastic lymphoma kinase
MET: Mesenchymal epithelial transition
KEAP1: Kelch-like ECH-associated protein 1
NFE2L2: Nuclear factor erythroid-2-related factor-2
EMT: Epithelial-mesenchymal transition
TCR: T cell receptor
cCRT: Concurrent chemo-radiotherapy
MDSCs: Myeloid-derived suppressor cells
EVs: Extracellular vesicles
DCs: Dendritic cells
CAMLs: Circulating cancer-associated macrophage-like cells
TAM: Tumor associated macrophage
CTECs: Circulating tumor endothelial cells
NLR: Neutrophil to lymphocyte ratio
ATB: Antibiotics
